# Circulation of the Nervous Fluid

**Published:** 1859-04

**Authors:** 


### CIRCULATION OF THE NERVOUS FLUID.

From the Gazette des Hopitaux for 1858, page 136.

M. Flourens read a note on the *nervous circulation*, before the Academy of Sciences, March 15, 1858. It is worthy of especial attention, as being probably the *point of departure* for a series of discoveries on the direction of the nervous currents.

"Recurrent Sensibility.—On a recent occasion, I recalled the beautiful experiment of Magendie on recurrent sensibility. If we cut the anterior root of a nerve, this root, which previously gave signs of sensibility through its whole extent, now does so in its peripheric end only. The end attached to the spinal cord has become insensible."

"The sensibility of the anterior root comes, then, from the posterior root, and not from the cord." "Moreover, if leaving the anterior root intact, we cut the posterior, the sensibility of the anterior root is lost." "It is, then, doubly shown, that the sensibility of the anterior root comes from the posterior." "But, how does it come? Evidently by return, by a circuit, or half circuit; and this half circuit is made at a distance." "Magendie cut the whole nerve, the compound nerve formed by the junction of the two roots near the point of junction. He cut it four lines, six lines from this point, and the sensibility of the anterior point was equally lost."

"This return, then, occurs at a distance very far off, and by the extremities of the nerves, as the return of the blood from the arteries to the veins only takes place at the extremities of these vessels."

"This recurrent sensibility is the first feature (*trait*) of what I call the nervous circulation."

"Reflex Action.—Cut the head off an animal, pinch his foot or his tail, and he withdraws it. This is the fact so well studied by Marshall Hall. What passes in this case? The sensitive nerve from the point irritated has carried the impression to a corresponding point of the spinal cord; from this point the irritation is communicated to the motor nerve, and the foot, or the tail, is moved." "Reflex action thus understood, is the complement of the recurrent action. This takes place by the extremity of the nerves, as that by the spinal cord."

"These two half circuits, the recurrent and the reflex, complete the circuit, and constitute the entire circulation."

